# 3-Meth­oxy-4-methyl-1*H*-1,2,4-triazol-5(4*H*)-one monohydrate

**DOI:** 10.1107/S1600536812023380

**Published:** 2012-05-31

**Authors:** Wei Gao, Xin-Ling Wang, Jing Yang, Xue-Fen Wu

**Affiliations:** a School of Pharmacy, Henan University of Traditional Chinese Medicine, Zhengzhou 450008, People’s Republic of China

## Abstract

In the title hydrate, C_4_H_7_N_3_O_2_·H_2_O, all the non-H atoms lie on a crystallographic mirror plane. The H atoms of both methyl groups are disordered over two sets of sites. In the crystal, N—H⋯O_w_ and O_w_—H⋯O_k_ (w = water and k = ketone) hydrogen bonds link the components into (010) sheets.

## Related literature

For related structures, see: Jin *et al.* (2011 ▶); Liu & Liu (2011 ▶); Liu *et al.* (2011 ▶, 2012 ▶); Ustabaş *et al.* (2010) ▶. For bioactivity data, see Tan *et al.* (2012 ▶).
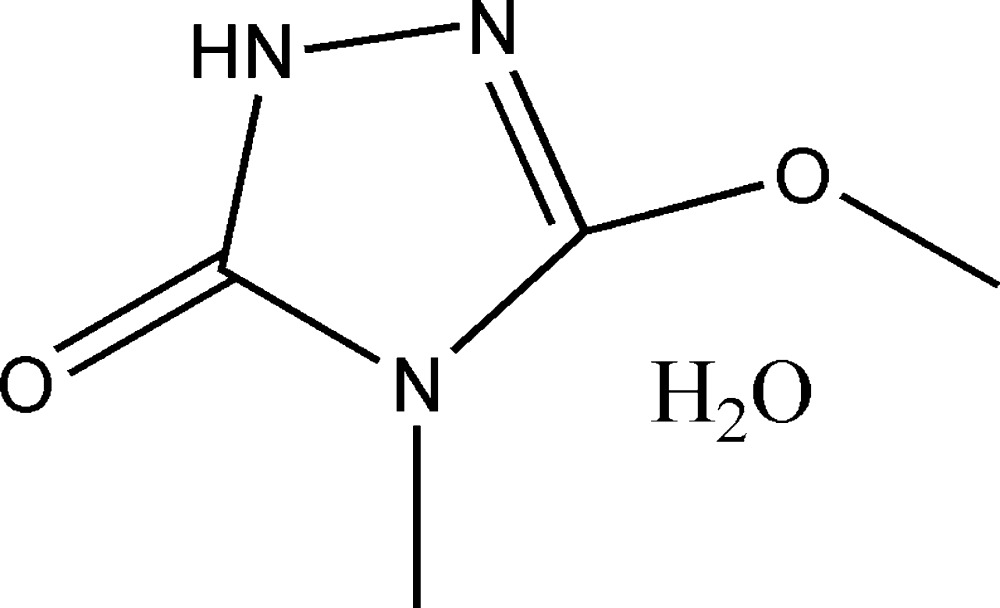


## Experimental

### 

#### Crystal data

C_4_H_7_N_3_O_2_·H_2_O
*M* *_r_* = 147.14
Orthorhombic, 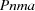

*a* = 6.810 (4) Å
*b* = 6.506 (4) Å
*c* = 15.277 (9) Å
*V* = 676.9 (7) Å^3^
*Z* = 4
Mo *K*α radiation
μ = 0.12 mm^−1^
*T* = 113 K
0.20 × 0.18 × 0.14 mm

#### Data collection

Rigaku Saturn724 CCD diffractometer
Absorption correction: multi-scan (*CrystalClear*; Rigaku/MSC, 2005 ▶) *T*_min_ = 0.976, *T*_max_ = 0.983
6611 measured reflections
873 independent reflections
727 reflections with *I* > 2σ(*I*)
*R*_int_ = 0.048

#### Refinement

*R*[*F* ^2^ > 2σ(*F* ^2^)] = 0.032
*wR*(*F* ^2^) = 0.090
*S* = 1.01
873 reflections
72 parameters
4 restraints
H atoms treated by a mixture of independent and constrained refinement
Δρ_max_ = 0.23 e Å^−3^
Δρ_min_ = −0.26 e Å^−3^

### 

Data collection: *CrystalClear* (Rigaku/MSC, 2005 ▶); cell refinement: *CrystalClear*; data reduction: *CrystalClear*; program(s) used to solve structure: *SHELXS97* (Sheldrick, 2008 ▶); program(s) used to refine structure: *SHELXL97* (Sheldrick, 2008 ▶); molecular graphics: *SHELXTL* (Sheldrick, 2008 ▶); software used to prepare material for publication: *CrystalStructure* (Rigaku/MSC, 2005 ▶).

## Supplementary Material

Crystal structure: contains datablock(s) global, I. DOI: 10.1107/S1600536812023380/hb6806sup1.cif

Structure factors: contains datablock(s) I. DOI: 10.1107/S1600536812023380/hb6806Isup2.hkl

Supplementary material file. DOI: 10.1107/S1600536812023380/hb6806Isup3.cml

Additional supplementary materials:  crystallographic information; 3D view; checkCIF report

## Figures and Tables

**Table 1:** Hydrogen-bond geometry (Å, °)

*D*—H⋯*A*	*D*—H	H⋯*A*	*D*⋯*A*	*D*—H⋯*A*
N2—H2⋯O3	0.90 (1)	1.85 (1)	2.7520 (15)	174 (1)
O3—H3*A*⋯O1^i^	0.87 (1)	1.89 (1)	2.7518 (18)	174 (1)
O3—H3*B*⋯O1^ii^	0.86 (1)	1.94 (1)	2.8024 (18)	179 (1)

Symmetry codes: (i) 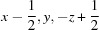; (ii) 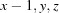.

**Fig. 1.**
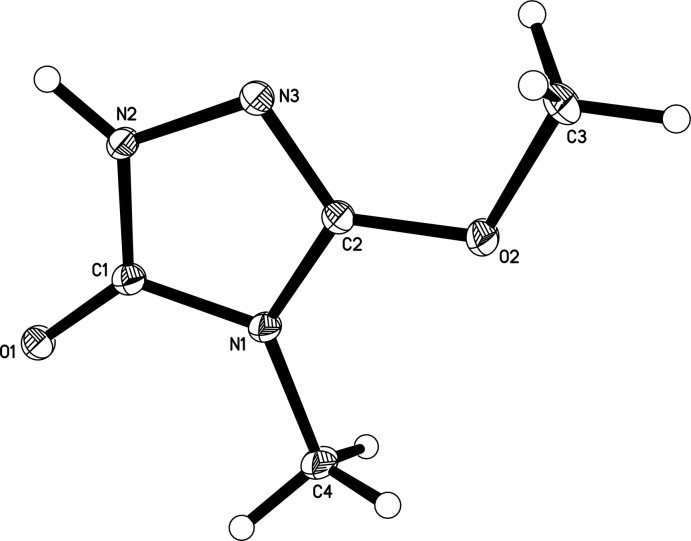
The molecular structure of (I). Displacement ellipsoids are drawn at the 30% probability level and H atoms are shown as small spheres of arbitrary radii.

**Fig. 2.**
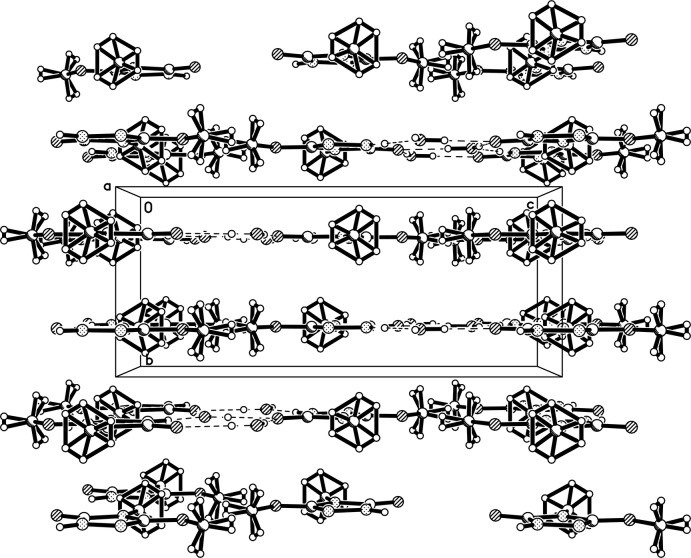
The crystal packing for (I).

## supplementary crystallographic information

### Comment

Sulfur and nitrogen heterocyclic compounds have received considerable attention in recent years because of their medicinal and pesticidal importance, such as 1,3,4-thiadiazoles, pyrimidines, 1,2,4-triazoles (Jin *et al.* 2011; Liu & Liu, 2011; Liu *et al.* 2011; Liu *et al.*, 2012; Tan *et al.*, 2011; Ustabaş *et al.*, 2010).

Single-crystal X-ray diffraction analysis reveals that the title compound crystallizes in the orthorhombic space group Pnma. As shown in Fig. 2, the crystal structure features intermolecular hydrogen bonds O-H···O and N-H···O.

### Experimental

The tite compound was available commercially. The crystals were grown from ethanol as colourless prisms

### Refinement

All the H atoms were positioned geometrically (C—H = 0.93–0.97 Å) and refined as riding with *U*_iso_(H) = 1.2*U*_eq_(C) or 1.5*U*_eq_(methyl C).

### Crystal data

**Table:**

C_4_H_7_N_3_O_2_·H_2_O	*D*_x_ = 1.444 Mg m^−^^3^
*M**_r_* = 147.14	Mo *K*α radiation, λ = 0.71073 Å
Orthorhombic, *P**n**m**a*	Cell parameters from 2353 reflections
*a* = 6.810 (4) Å	θ = 3.0–27.8°
*b* = 6.506 (4) Å	µ = 0.12 mm^−^^1^
*c* = 15.277 (9) Å	*T* = 113 K
*V* = 676.9 (7) Å^3^	Prism, colorless
*Z* = 4	0.20 × 0.18 × 0.14 mm
*F*(000) = 312	

### Data collection

**Table:**

Rigaku Saturn724 CCD diffractometer	873 independent reflections
Radiation source: rotating anode	727 reflections with *I* > 2σ(*I*)
Multilayer monochromator	*R*_int_ = 0.048
Detector resolution: 14.22 pixels mm^-1^	θ_max_ = 27.9°, θ_min_ = 3.3°
ω and φ scans	*h* = −8→8
Absorption correction: multi-scan (*CrystalClear*; Rigaku/MSC, 2005)	*k* = −8→7
*T*_min_ = 0.976, *T*_max_ = 0.983	*l* = −20→20
6611 measured reflections	

### Refinement

**Table:**

Refinement on *F*^2^	Primary atom site location: structure-invariant direct methods
Least-squares matrix: full	Secondary atom site location: difference Fourier map
*R*[*F*^2^ > 2σ(*F*^2^)] = 0.032	Hydrogen site location: inferred from neighbouring sites
*wR*(*F*^2^) = 0.090	H atoms treated by a mixture of independent and constrained refinement
*S* = 1.01	*w* = 1/[σ^2^(*F*_o_^2^) + (0.0616*P*)^2^] where *P* = (*F*_o_^2^ + 2*F*_c_^2^)/3
873 reflections	(Δ/σ)_max_ = 0.003
72 parameters	Δρ_max_ = 0.23 e Å^−^^3^
4 restraints	Δρ_min_ = −0.26 e Å^−^^3^

### Special details

**Table:**

Geometry. All e.s.d.'s (except the e.s.d. in the dihedral angle between two l.s. planes) are estimated using the full covariance matrix. The cell e.s.d.'s are taken into account individually in the estimation of e.s.d.'s in distances, angles and torsion angles; correlations between e.s.d.'s in cell parameters are only used when they are defined by crystal symmetry. An approximate (isotropic) treatment of cell e.s.d.'s is used for estimating e.s.d.'s involving l.s. planes.
Refinement. Refinement of *F*^2^ against ALL reflections. The weighted *R*-factor *wR* and goodness of fit *S* are based on *F*^2^, conventional *R*-factors *R* are based on *F*, with *F* set to zero for negative *F*^2^. The threshold expression of *F*^2^ > σ(*F*^2^) is used only for calculating *R*-factors(gt) *etc*. and is not relevant to the choice of reflections for refinement. *R*-factors based on *F*^2^ are statistically about twice as large as those based on *F*, and *R*- factors based on ALL data will be even larger.

### Fractional atomic coordinates and isotropic or equivalent isotropic displacement parameters (Å^2^)

**Table:**

	*x*	*y*	*z*	*U*_iso_*/*U*_eq_	Occ. (<1)
O1	0.64561 (9)	0.2500	0.35699 (4)	0.02165 (15)	
O2	0.49415 (9)	0.2500	0.65038 (4)	0.02269 (16)	
N1	0.62148 (10)	0.2500	0.50977 (4)	0.01766 (17)	
N2	0.35229 (11)	0.2500	0.43609 (4)	0.01895 (17)	
N3	0.29539 (11)	0.2500	0.52418 (5)	0.01921 (18)	
C1	0.54905 (11)	0.2500	0.42578 (6)	0.0172 (2)	
C2	0.46308 (12)	0.2500	0.56483 (6)	0.0169 (2)	
C4	0.82847 (13)	0.2500	0.53342 (6)	0.0235 (2)	
H4A	0.9060	0.1949	0.4847	0.035*	0.50
H4B	0.8481	0.1642	0.5854	0.035*	0.50
H4C	0.8707	0.3909	0.5461	0.035*	0.50
C3	0.31615 (14)	0.2500	0.70293 (6)	0.0257 (2)	
H3D	0.2528	0.1151	0.6990	0.039*	0.50
H3E	0.2262	0.3558	0.6811	0.039*	0.50
H3C	0.3495	0.2791	0.7641	0.039*	0.50
O3	0.04758 (10)	0.2500	0.31776 (5)	0.0449 (2)	
H2	0.2590 (12)	0.2500	0.3942 (6)	0.034 (3)*	
H3A	0.0698 (13)	0.2500	0.2619 (4)	0.047 (4)*	
H3B	−0.0752 (9)	0.2500	0.3306 (6)	0.063 (4)*	

### Atomic displacement parameters (Å^2^)

**Table:**

	*U*^11^	*U*^22^	*U*^33^	*U*^12^	*U*^13^	*U*^23^
O1	0.0167 (3)	0.0331 (3)	0.0152 (3)	0.000	0.0026 (2)	0.000
O2	0.0164 (3)	0.0380 (4)	0.0136 (3)	0.000	0.0014 (2)	0.000
N1	0.0116 (3)	0.0256 (4)	0.0158 (3)	0.000	0.0001 (3)	0.000
N2	0.0134 (3)	0.0301 (4)	0.0133 (3)	0.000	−0.0005 (3)	0.000
N3	0.0152 (3)	0.0273 (4)	0.0151 (3)	0.000	0.0014 (3)	0.000
C1	0.0154 (4)	0.0183 (4)	0.0179 (4)	0.000	−0.0004 (3)	0.000
C2	0.0149 (4)	0.0211 (4)	0.0147 (4)	0.000	0.0011 (3)	0.000
C4	0.0116 (4)	0.0377 (5)	0.0213 (4)	0.000	−0.0012 (3)	0.000
C3	0.0212 (4)	0.0394 (5)	0.0166 (4)	0.000	0.0074 (3)	0.000
O3	0.0155 (3)	0.1026 (7)	0.0165 (3)	0.000	−0.0008 (3)	0.000

### Geometric parameters (Å, º)

**Table:**

O1—C1	1.2397 (12)	N3—C2	1.2999 (12)
O2—C2	1.3239 (13)	C4—H4A	0.9800
O2—C3	1.4540 (13)	C4—H4B	0.9800
N1—C2	1.3679 (12)	C4—H4C	0.9800
N1—C1	1.3746 (13)	C3—H3D	0.9800
N1—C4	1.4552 (14)	C3—H3E	0.9800
N2—C1	1.3492 (13)	C3—H3C	0.9800
N2—N3	1.4003 (12)	O3—H3A	0.867 (6)
N2—H2	0.901 (7)	O3—H3B	0.859 (6)
			
C2—O2—C3	114.32 (7)	N1—C4—H4A	109.5
C2—N1—C1	106.92 (8)	N1—C4—H4B	109.5
C2—N1—C4	127.68 (8)	H4A—C4—H4B	109.5
C1—N1—C4	125.41 (7)	N1—C4—H4C	109.5
C1—N2—N3	112.77 (7)	H4A—C4—H4C	109.5
C1—N2—H2	128.1 (6)	H4B—C4—H4C	109.5
N3—N2—H2	119.1 (6)	O2—C3—H3D	109.5
C2—N3—N2	102.47 (7)	O2—C3—H3E	109.5
O1—C1—N2	128.74 (8)	H3D—C3—H3E	109.5
O1—C1—N1	126.94 (8)	O2—C3—H3C	109.5
N2—C1—N1	104.32 (7)	H3D—C3—H3C	109.5
N3—C2—O2	127.74 (8)	H3E—C3—H3C	109.5
N3—C2—N1	113.52 (9)	H3A—O3—H3B	113.2 (8)
O2—C2—N1	118.75 (8)		
			
C1—N2—N3—C2	0.0	N2—N3—C2—N1	0.0
N3—N2—C1—O1	180.0	C3—O2—C2—N3	0.0
N3—N2—C1—N1	0.0	C3—O2—C2—N1	180.0
C2—N1—C1—O1	180.0	C1—N1—C2—N3	0.0
C4—N1—C1—O1	0.0	C4—N1—C2—N3	180.0
C2—N1—C1—N2	0.0	C1—N1—C2—O2	180.0
C4—N1—C1—N2	180.0	C4—N1—C2—O2	0.0
N2—N3—C2—O2	180.0		

### Hydrogen-bond geometry (Å, º)

**Table:**

*D*—H···*A*	*D*—H	H···*A*	*D*···*A*	*D*—H···*A*
N2—H2···O3	0.90 (1)	1.85 (1)	2.7520 (15)	174 (1)
O3—H3*A*···O1^i^	0.87 (1)	1.89 (1)	2.7518 (18)	174 (1)
O3—H3*B*···O1^ii^	0.86 (1)	1.94 (1)	2.8024 (18)	179 (1)

Symmetry codes: (i) *x*−1/2, *y*, −*z*+1/2; (ii) *x*−1, *y*, *z*.
